# Assessment of health-related quality of life in individuals with depressive symptoms: validity and responsiveness of the EQ-5D-3L and the SF-6D

**DOI:** 10.1007/s10198-022-01543-w

**Published:** 2022-11-16

**Authors:** Maike Stolz, Christian Albus, Manfred E. Beutel, Hans-Christian Deter, Kurt Fritzsche, Christoph Herrmann-Lingen, Matthias Michal, Katja Petrowski, Joram Ronel, Jobst-Hendrik Schultz, Wolfgang Söllner, Cora Weber, Martina de Zwaan, Christian Krauth

**Affiliations:** 1 Institute of Epidemiology Social Medicine and Health System Research, Hannover Medical School, Hanover, Germany; 2 Center for Health Economics Research Hanover (CHERH), Hanover, Germany; 3 Department of Psychosomatics and Psychotherapy, University of Cologne, Cologne, Germany; 4 Department of Psychosomatic Medicine and Psychotherapy, University Medical Center Mainz, Mainz, Germany; 5 Department of Psychosomatics and Psychotherapy, Charité Universitaetsmedizin Berlin, Campus Benjamin Franklin, Berlin, Germany; 6 Faculty of Medicine, Department of Psychosomatic Medicine and Psychotherapy, Medical Center-University of Freiburg, Freiburg, Germany; 7 Department of Psychosomatic Medicine and Psychotherapy, University of Göttingen Medical Center, Göttingen, Germany; 8 German Center for Cardiovascular Research (DZHK), Partner Site Göttingen, Göttingen, Germany; 9 Department of Psychotherapy and Psychosomatics, Technical University of Dresden, Dresden, Germany; 10 Department of Psychosomatic Medicine and Psychotherapy, Clinic Barmelweid, Barmelweid, Switzerland; 11 Department of Psychosomatic Medicine and Psychotherapy, University Hospital Rechts Der Isar, Technische Universitaet München, Munich, Germany; 12 Department of General Internal Medicine and Psychosomatics, Heidelberg University Hospital, Heidelberg, Germany; 13 Department of Psychosomatic Medicine and Psychotherapy, Paracelsus Medical University Nuremberg, Nuremberg, Germany; 14 Department of Psychosomatic Medicine and Psychotherapy, Oberhavel Clinic, Clinic Hennigsdorf, Hennigsdorf, Germany; 15 Department of Psychosomatic Medicine and Psychotherapy, Hannover Medical School, Hanover, Germany

**Keywords:** EQ-5D-3L, SF-6D, Mental disorder, Validity, Responsiveness, I100

## Abstract

**Background:**

The EQ-5D and the SF-6D are examples of commonly used generic preference-based instruments for assessing health-related quality of life (HRQoL). However, their suitability for mental disorders has been repeatedly questioned.

**Objective:**

To assess the responsiveness and convergent validity of the EQ-5D-3L and SF-6D in patients with depressive symptoms.

**Methods:**

The data analyzed were from cardiac patients with depressive symptoms and were collected as part of the SPIRR-CAD (Stepwise Psychotherapy Intervention for Reducing Risk in Coronary Artery Disease) trial. The EQ-5D-3L and SF-6D were compared with the HADS (Hospital Anxiety and Depression Scale) and PHQ-9 (Patient Health Questionnaire) as disease-specific instruments. Convergent validity was assessed using Spearman’s rank correlation. Effect sizes were calculated and ROC analysis was performed to determine responsiveness.

**Results:**

Data from 566 patients were analysed. The SF-6D correlated considerably better with the disease-specific instruments (|*r*_s_|= 0.63–0.68) than the EQ-5D-3L (|*r*_s_|= 0.51–0.56). The internal responsiveness of the SF-6D was in the upper range of a small effect (ES: − 0.44 and − 0.47), while no effect could be determined for the EQ-5D-3L. Neither the SF-6D nor the EQ-5D-3L showed acceptable external responsiveness for classifying patients’ depressive symptoms as improved or not improved. The ability to detect patients whose condition has deteriorated was only acceptable for the EQ-5D-3L.

**Conclusion:**

Overall, both the convergent validity and responsiveness of the SF-6D are better than those of the EQ-5D-3L in patients with depressive symptoms. The SF-6D appears, therefore, more recommendable for use in studies to evaluate interventions for this population.

**Supplementary Information:**

The online version contains supplementary material available at 10.1007/s10198-022-01543-w.

## Background

Depressive disorders are of great importance to society due to their burden of disease, prevalence, frequent recurrence or long-lasting course, increased use of the health care system and the associated direct and indirect costs [1]. In terms of disability adjusted life years (DALYs), the burden of disease of depressive disorders was in third place in a worldwide comparison of all illnesses in 2001 in high-income countries [2]. According to a prognosis by the World Health Organisation (WHO), depressive disorders will be the most significant of the widespread diseases that impair or shorten life by the year 2030. Since the years of life lost due to premature death are of little significance in depression, it becomes clear how severely the way of life is impaired by this illness [2, 3]. For the individual concerned, the presence of depressive symptoms is associated with a loss of health-related quality of life (HRQoL) by influencing the physical, emotional and social aspects of well-being [4, 5].

The EQ-5D and SF-6D are generic multi-attribute health status classification systems, which are used to assess HRQoL in health economic evaluations [6, 7]. By evaluating health states according to their relative value (derived from preferences), and summarizing them into a single index value (utility value). They are a widely used because of being an indirect alternative for measuring preferences using simple questionnaires, as measuring preferences through direct questioning and assessment by the patient concerned is very time-consuming and complex. [8–11].

Results of health economic evaluations are part of allocation decisions of limited resources in the health care system. A prerequisite for a reliable comparison of different interventions and a resulting “fair” allocation is the suitability of the health economic quality-of-life instruments in the context of different diseases and populations [8].

However, the suitability of generic instruments for assessing HRQoL in mental disorders has repeatedly been questioned [12–18]. The main concerns are based on the design of these instruments with a focus on physical complaints, so that (changes in the) psychological components are not sufficiently taken into account in the summary scores and the index scores [15–18]. This seems to be especially true for the EQ-5D, as four of the five dimensions are in the physical domain, while the six dimensions of the SF-6D are balanced between the physical and psychological domains [19]. In general, it is often discussed that responsiveness of generic instruments is lower than that of disease-specific instruments because the questions are less specific to the symptoms of the underlying disease and therefore minor changes are not captured. However, the generality of this statement is controversial [8, 20, 21].

The purpose of this study was to evaluate whether the EQ-5D-3L and SF-6D, as examples of commonly used generic preference-based instruments for assessing HRQoL, are suitable for patients with depressive symptoms and whether either instrument is superior to the other for this purpose. To assess the responsiveness and convergent validity of the EQ-5D-3L and SF-6D, they were compared to the depression scales of the disease-specific Hospital Anxiety and Depression Scale (HADS) and Patient Health Questionnaire (PHQ-9). The following hypotheses were examined:

1. The correlation between the EQ-5D-3L and disease-specific instruments differs from the correlation between the SF-6D and disease-specific instruments.
2. The responsiveness of the generic instruments differs from the responsiveness of the disease-specific instruments.
3. There is a difference in the responsiveness of the EQ-5D-3L and SF-6D.

## Methods

The analyses carried out are based on data from the Stepwise Psychotherapy Intervention for Reducing Risk in Coronary Artery Disease (SPIRR-CAD) study. Details and results of the randomized controlled trial are described elsewhere [22, 23]. Briefly, the SPIRR-CAD study was designed to test the hypothesis that a stepwise psychotherapy intervention is more effective in mitigating depressive symptoms in cardiac patients than one information session added to usual care. Inclusion criteria included age between 18 and 75 years, documented coronary artery disease (CAD) and a depression score higher than 7 points on the HADS depression scale. Exclusion criteria included inability to speak German, severe heart failure (New York Heart Association (NYHA) Class IV), scheduled cardiac surgery within the next 3 months, severe depressive episode according to the Structured Clinical Interview for DSM-IV or other severe or life-threatening physical or mental illness. All patients received usual care by their general practitioner and/or cardiologist. Patients in the control group additionally received one information session of 30 to 45 min providing information about healthy behaviours and psychosocial factors in CAD. Patients in the intervention group were offered three individual psychotherapy sessions. All patients were reassessed with the HADS depression scale, and only those continue to show depressive symptoms (HADS score > 7) after 4 to 8 weeks were offered 25, 90-min sessions of group psychotherapy.

### Instruments

Various survey instruments were used in the SPIRR-CAD study. The SF-6D (SF-36), EQ-5D-3L, HADS and PHQ-9 were available for the comparison of generic and disease-specific instruments in depressive disorders.

The HADS depression scale is a psychometric self-assessment tool to measure depressive symptoms in patients with primary somatic diseases [24, 25]. It consists of seven items each rated from 0 to 3 according to severity of difficulty experienced. Total score ranges from 0 (no depression) to 21, in which ≤ 7 points are considered unremarkable, 8–10 points are considered reflecting marginal depression and ≥ 11 points are considered conspicuous.

The Patient Health Questionnaire 9-item (PHQ-9) is a self-assessment depression screening tool for administration among adults in primary care settings [26, 27]. It consists of nine items each rated from 0 to 3 according to frequency of occurrence. Total score ranges from 0 (no depression) to 27, in which ≤ 4 points are assessed as no depressive symptoms, 5–9 points as mild or moderate depressive symptoms and ≥ 10 points as suggestive of major depression.

The SF-6D is a generic preference-based index instrument, developed for use in health economic evaluation studies. It can be derived from data from the SF-36, which is one of the most widely used generic HRQoL instruments worldwide [6]. The SF-6D consists of eleven items (of the SF-36) that are divided into six dimensions: physical functioning, role limitations, social functioning, pain, mental health and vitality. Each dimension has between two and six levels. A SF-6D health state is defined by selecting one level from each dimension resulting in 18,000 different possible health states. In the end, every health state can be described by an index value. Therefore, a representative sample of the general population has to assess selected health states using preference-based methods (e.g. standard gamble or time trade off). A value set, weighting the levels in each dimension, is calculated from the results using multiple regression analyses. This value set can be used to calculate a single index value out of the data derived from an applied SF-36 questionnaire.

The EQ-5D is a generic preference-based index instrument for describing, quantifying and valuing HRQoL [7]. It comprises five dimensions: mobility, self-care, usual activities, pain/discomfort and anxiety/depression. Each dimension of the EQ-5D-3L used in this study has three levels. An EQ-5D-3L health state is defined by selecting one level from each dimension resulting in 243 different possible health states [28]. A single index value can then be derived from the characterized health state using the method described above for the SF-6D.

The choice of a value set can affect the resulting index value, because value sets are generally meant to reflect the preferences of specific countries which can be different from each other [29]. As there are no values for the German context, it was decided to use the British tariff for the SF-6D (SF-6D_UK_) [6]. Although there is a German value set for the EQ-5D-3L (EQ-5D-3L_GER_) [30], the main analyses to test hypotheses were carried out using the UK tariff (EQ-5D-3L_UK_) [31, 32] for two reasons. (1) A comparison of the suitability of the EQ-5D-3L and SF-6D for people with depressive symptoms is more valid when the underlying preferences are from the population of the same country. (2) The German tariff does not contain a discount value for mild depression. This means that when comparing responsiveness, a change between mild and non-existent depression cannot be mapped. However, since the German tariff for the EQ-5D-3L should not be completely ignored in the context of a German study population, the results of the analyses are also presented for the EQ-5D-3L_GER_.

### Data analysis

All analyses were carried out using SPSS version 22 and cocor web interface [33].

To investigate whether the EQ-5D-3L and SF-6D are appropriate for use with patients with depressive symptoms, convergent validity and responsiveness were examined.

The convergent validity describes the degree to which two measures of constructs that theoretically should be correlated, are in fact correlated [34]. In this analysis the index scores of the EQ-5D-3L indices and SF-6D_UK_ were compared to the sum scores of the disease-specific HADS and PHQ-9. Spearman rank correlation coefficients (*r*_s_) of the scores at 6 months were calculated to build a correlation matrix.

To test hypothesis 1, correlation coefficients of the EQ-5D-3L_UK_, SF-6D_UK_, HADS and PHQ-9 have to be compared and a test of significance was necessary to control for possible differences occurring by chance [33, 35]. Dunn and Clark’s z was chosen because of its appropriateness for dependent correlations with either overlapping or nonoverlapping variables [33, 36, 37]. The analyses were carried out using the cocor web interface [33].

Responsiveness is defined as the ability of an instrument to detect change over time [38]. Internal responsiveness was assessed using effect sizes [39]. Since there is controversy regarding the most appropriate effect size for calculating responsiveness [39], both the standardized effect size (SES) and the standardized response mean (SRM) were used:

$SES= \frac{{M}_{t1}-{M}_{t2}}{{SD}_{t1}}$ and $SRM= \frac{{M}_{t1}-{M}_{t2}}{{SD}_{t2-t1}}$,where “${M}_{t1}$” is the arithmetic mean at baseline assessment, “${M}_{t2}$”” is the arithmetic mean at 18 months, “${SD}_{t1}$” is the standard deviation (SD) at baseline assessment and ${SD}_{t2-t1}$ is the standard deviation (SD) of the measured difference between baseline assessment and assessment after 18 months. A clinically relevant change of at least two points on the HADS is an indicator of an improvement/deterioration and is used as a reference criterion [40].

So far, there are no specific benchmarks for effect sizes as a measure of responsiveness. For this reason, the “rule of thumb” according to Cohen is often used to assess effect sizes in intervention studies [41]. This means that a value between 0.2 and 0.49 corresponds to a small effect, a value between 0.5 and 0.79 corresponds to a medium effect and a value of > 0.8 corresponds to a large effect [42–44].

To test hypotheses 2 and 3 the Modified Jacknife Test was used as a test of significance [45–47]. This test is based on a linear regression, where the dependent variable contains the difference of the SES/SRM between the two instruments to be compared, while the independent variable consists of the “centered SES/SRM”. The “centered SES/SRM” is formed by subtracting the mean SES/SRM of one of the two instruments to be compared (which one is not relevant) from the individual SES/SRM for each patient. A significant intercept coefficient represents a significant difference between the SES/SRM of the two scales to be compared [46, 47]. To control for possible violation of requirements for linear regression (normal distribution of the residues and homoscedasticity) the bootstrap method was carried out. The intercept coefficient and the associated Modified Jacknife* Test* are only considered significant if the confidence intervals generated by bootstrapping do not contain the value “0”.

External responsiveness was assessed using Receiver operating characteristics (ROC) curves and the area under the curve (AUC) as a reference number [39, 48, 49]. In this context, responsiveness is described in terms of change sensitivity and change specificity. “Change sensitivity” means the probability of the instrument correctly classifying patients who demonstrate a change on an external criterion, whereas “change specificity” means the probability of an instrument correctly classifying patients who do not demonstrate change on an external criterion. A change can either mean an improvement or a deterioration. Separate ROC curves must be calculated for both cases [49]. As an external criterion for change, the HADS was used. Here, a change by two points between baseline assessment and follow-up assessment after 18 months was defined as the Minimal Clinical Important Difference (MCID) [40] and accordingly assigned to the status “changed”. If there was a difference of less than two points, the status was considered as being “unchanged”.

The AUC represents the probability that an instrument correctly classifies patients as improved or not improved and deteriorated or not deteriorated, respectively [39, 48]. An AUC of 0.5 means that an instrument cannot discriminate between patients whose status has changed and Patients whose status has not changed, while a value of 1.0 corresponds to perfect discriminatory power. A value ≥ 0.7 is considered moderate [50].

The formulated hypotheses are global hypotheses, which have to be proved by multiple statistical tests. To avoid the error of multiple comparisons a Bonferroni correction was conducted. Each single test was evaluated with a corrected α level (α’) with

$$\alpha^{\prime} = \alpha /k,$$

where ‘*α*’ is the critical probability (*p*) level and ‘*k*’ is the number of tests performed [51]. The assumed *α* level of 0.05 for each single test was therefore corrected to an *α*’ of 0.0125 for hypothesis 1 (for *k* = 4), to an *α*’ of 0.00625 (for *k* = 8) for hypothesis 2 and to an *α*’ of 0.025 (for *k* = 2) for hypothesis 3 (see Supplementary Information).

## Results

The cohort consisted of 566 patients whose detailed characteristics have been reported elsewhere [23]. The mean age was 59.2 years and 21.1% were female. Most patients (81.7%) were classified in NYHA class I or II. Overall, 11.6% of the patients received antidepressant medication and 11.1% were in psychotherapy within the preceding 12 months.

### Descriptive analysis

Means and medians of the compared instruments are shown in Table 1. With a mean score of 10.42 on the baseline measure on the HADS and 9.95 on the PHQ-9, participants in the study had mild to moderate depressive symptoms on average. The mean index value of the SF-6D_UK_ was at least 0.03 points lower than that of the EQ-5D-3L_UK_ at all three measurement points. In addition, the standard deviation of the SF-6D_UK_ was only half as large as that of the EQ-5D-3L_UK_. Noticeably, the mean value of the EQ-5D-3L_UK_ was significantly lower than that of the EQ-5D-3L_GER_, with a difference of at least 0.13 points_._

**Table 1:** Means and medians of individual instruments at three central measurement points

		Baseline	6 months	18 months
EQ-5D-3L_GER_	*n*	521	436	384
	Mean (SD)	0.79 (0.22)	0.80 (0.23)	0.81 (0.24)
	Median (IQR)	0.89 (0.79-0.89)	0.89 (0.79-0.89)	0.89 (0.79-1.00)
EQ-5D-3L_UK_	*n*	521	436	384
	Mean (SD)	0.64 (0.26)	0.67 (0.28)	0.68 (0.28)
	Median (IQR)	0.71 (0.62-0.80)	0.73 (0.62-0.80)	0.73 (0.66-0.85)
SF-6D_UK_	*n*	475	425	355
	Mean (SD)	0.60 (0.11)	0.64 (0.12)	0.65 (0.12)
	Median (IQR)	0.60 (0.53-0.65)	0.62 (0.56-0.73)	0.64 (0.56-0.74)
HADS	*n*	566	445	397
	Mean (SD)	10.42 (2.54)	8.98 (3.90)	8.13 (3.94)
	Median (IQR)	10.00 (8.00-12.00)	9.00 (6.00-12.00)	8.00 (6.00-11.00)
PHQ-9	*n*	526	446	387
	Mean (SD)	9.95 (5.27)	9.13 (5.03)	8.29 (5.15)
	Median (IQR)	9.00 (6.00-13.00)	9.00 (5.00-13.00)	7.00 (4.00-11.00)

*n* sample size, *SD* standard deviation, *IQR* interquartile range

Medians and means were close for most instruments. Clear differences can only be seen in the EQ-5D-3L_UK_ and EQ-5D-3L_GER_. The difference between the SF-6D_UK_ and the EQ-5D-3L_UK_ was more obvious when looking at the median, with differences between 0.08 and 0.11 points, than for the mean differences.

### Convergent validity

A higher score on the generic instruments equates to a better state of health, whereas a higher score on the disease-specific instruments is associated with a more severe disorder. As expected, this results in a positive correlation between the generic and disease-specific instruments among each other and a negative correlation between the generic and disease-specific instruments (Table 2). The SF-6D_UK_ correlates best with the EQ-5D-3L_UK_, while the EQ-5D-3L_UK_ is more strongly associated with the EQ-5D-3L_GER_. Overall, the SF-6D_UK_ correlates considerably better with the disease-specific instruments (|*r*_s_|= 0,63–0,68) than the EQ-5D-3L_UK_ (|*r*_s_|= 0,51–0,56) or EQ-5D-3L_GER_ (|*r*_s_|= 0,42–0,45). The comparison of the correlation coefficients of the SF-6D_UK_ with the disease-specific instruments and the EQ-5D-3L_UK_ with the disease-specific instruments could confirm that the differences found were significantly different and in favour of the SF-6D (see Supplementary Information, Table 1). Therefore, it can be assumed that the SF-6D_UK_ shows a higher convergent validity for use in people with depressive symptoms than the EQ-5D-3L_UK_, which confirms hypothesis 1.

**Table 2:** Correlation matrix of all instruments at T2

		EQ-5D-3L_GER_	EQ-5D-3L_UK_	SF-6D_UK_	HADS	PHQ-9
EQ-5D-3L_GER_	n	436	436	405	425	428
	r_s_	1.00	0.93*	0.65*	− 0.42*	− 0.45*
EQ-5D-3L_UK_	n		436	405	425	428
	r_s_		1.00	0.72*	− 0.51*	− 0.56*
SF-6D_UK_	n			425	412	416
	r_s_			1.00	− 0.63*	− 0.68*
HADS	n				445	436
	r_s_				1.00	0.71*
PHQ-9	n					449
	r_s_					1.00

*n* sample size, *r*_*s*_ Spearman rank correlation coefficient

*p < 0.01

### Internal responsiveness

The SF-6D_UK_ reached values between − 0.44 and − 0.47 in the upper range of a small effect, while no effect can be demonstrated for the EQ-5D-3L indices, equivalent to a non-existent sensitivity to change for the studied population (Table 3). Using the Modified Jacknife Test, it was possible to determine that the differences in responsiveness were significant, confirming that the SF-6D_UK_ is more sensitive to change in depressive symptoms than the EQ-5D-3L_UK_ (see Supplementary Information, Table 2). The picture was also heterogeneous for the disease-specific instruments. While the HADS was the most responsive instrument with medium to large effects, only small effects could be achieved for the PHQ-9 (0.31–0.36), which were even smaller than those of the SF-6D. Since all multiple comparisons between the generic and disease-specific instruments were significant, it must be stated that neither the generic nor the disease-specific instruments can be classified as being generally more responsive than the others (see Supplementary Information, Table 2).

**Table 3:** SES and SRM of the instruments

	SES	SRM
EQ-5D-3L_GER_	− 0.09	− 0.08
EQ-5D-3L_UK_	− 0.16	− 0.15
SF-6D_UK_	− 0.47	− 0.44
HADS	0.90	0.62
PHQ-9	0.31	0.36

Negative values represent an improvement for the generic instruments, whereas positive values represent an improvement for the HADS and PHQ-9

### External responsiveness

According to our current data, the ability to discriminate between patients who improved and those who did not improve cannot be considered as good or moderate for any of the here applied instruments (Table 4). Only the PHQ-9 can be classified as acceptable for detecting patients whose condition has improved based on the result of the HADS. The ability to detect patients whose condition had deteriorated was only acceptable for the EQ-5D-3L_UK_.

**Table 4:** Area under the curve of the inserted instruments

	Improvement AUC [95% CI]	Deterioration AUC [95% CI]
EQ-5D-3L_GER_	0.533 [0.462; 0.605]	0.587 [0.493; 0.682]
EQ-5D-3L_UK_	0.553 [0.482; 0.625]	0.626 [0.533; 0.718]
SF-6D_UK_	0.592 [0.524; 0.660]	0.572 [0.462; 0.682]
PHQ-9	0.634 [0.569; 0.699]	0.531 [0.431; 0.630]

AUC area under the curve, *CI* confidence interval

The ROC curves of improvement are based on *n* = 334 (*n* = 221 improved, *n* = 113 unchanged)

The ROC curves of deterioration are based on *n* = 176 (*n* = 63 deteriorated, *n* = 113 unchanged)

**Fig. 1:**
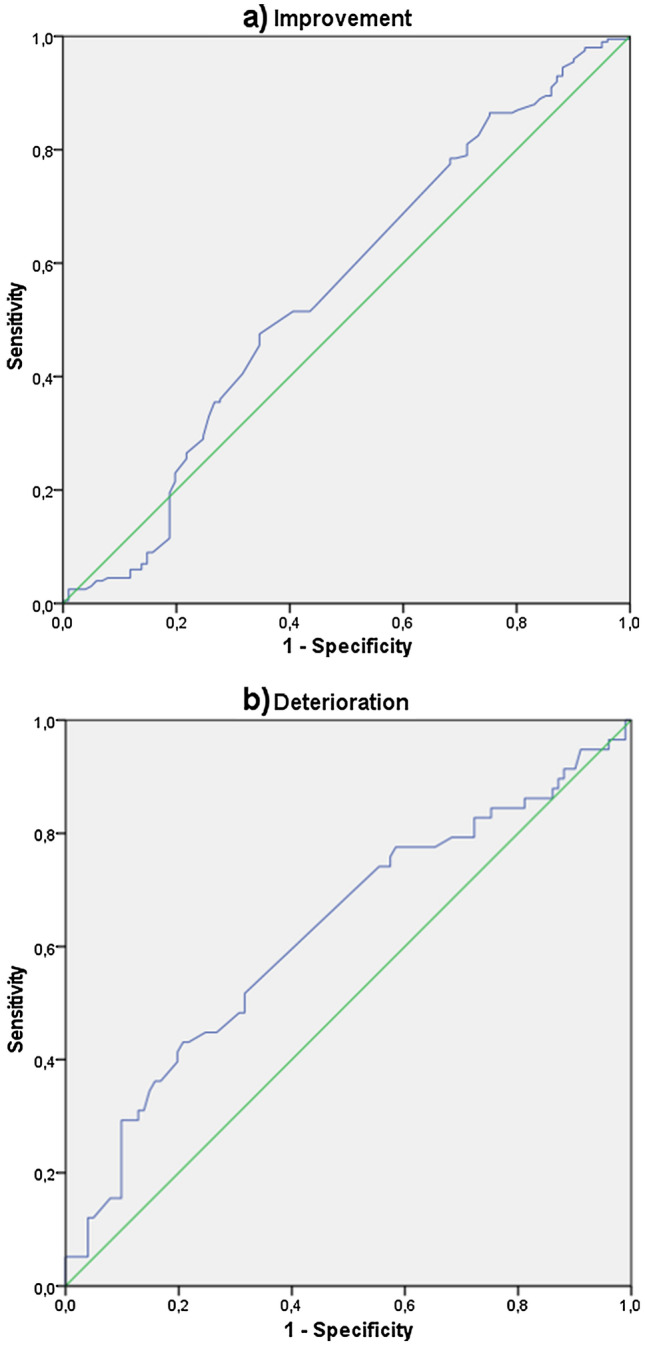
ROC curves of the EQ-5D-3L_UK_ for changes from T0 to T3

Figure 1 shows an example of the ROC curves of the EQ-5D-3L_UK_.

## Discussion

In the light of limited resources in the health care system and constantly rising costs resulting from demographic change as well as expensive innovations in modern and advanced health care, there is a need for improving conditions for evidence-based allocation decisions. Health economic methods can help to make the allocation of resources in the healthcare system quantifiable. The use and suitability of generic preference-based quality of life instruments is essential for a targeted evaluation of medical interventions. However, its use in assessing mental disorders is questioned. In this context, the EQ-5D-3L and SF-6D were tested for use in patients with depressive symptoms.

### Descriptive analysis

The mean baseline values for the SF-6D_UK_ and for the EQ-5D-3L_UK_ are comparable to those of other studies addressing depressive disorders that also used the British value sets used in this work. Sobocki et al. found an EQ-5D-3L_UK_ score of 0.60 for mild depression in their observational study of medicated depressed patients [52]. When comparing the EQ-5D-3L_UK_ and the SF-6D_UK_ using data from a multi-center RCT to evaluate different therapeutic approaches for depressive and anxiety disorders, both the EQ-5D-3L_UK_ and the SF-6D_UK_ for mild symptoms were 0.60 [53]. A further comparison showed an index of 0.62 for the EQ-5D-3L_UK_ and 0.63 for the SF-6D_UK_ for people with mild depression in a population sample in Canada [54].

Similar to the present work, Lamers et al. found that the mean and median for the EQ-5D-3L_UK_ differed significantly, while they were perfectly on top of each other for the SF-6D_UK_ [53]. This discrepancy can possibly be explained by the preference values of the individual health conditions. For the SF-6D_UK_, the worst health state is associated with a preference value of 0.296, while for the EQ-5D-3L_UK_, negative values (for health states considered worse than death) and a preference value of -0.594 in the worst case are also possible [6, 32]. Such “outliers” lead to inaccurate estimates of mean values, which is also reflected in the significantly larger standard deviation of the EQ-5D-3L_UK_ compared to the SF-6D_UK_ [53]. A lower standard deviation enables more precise estimates. This is particularly relevant if quality-adjusted life years (QALYs) for cost-effectiveness studies are calculated based on the index values, which in turn can be used to compare two interventions and influence allocation decisions [55].

### Convergent validity

The correlation between the HADS and the PHQ-9 for baseline measurement was in a similar range as in the study by Cameron and colleagues. The authors compared the two disease-specific instruments for use in primary care of patients with mild to moderate mental health problems and found a correlation coefficient of 0.68 [56].

A higher convergent validity was determined for the SF-6D_UK_ and the depression scales than for the EQ-5D-3L_UK_ as well as the EQ-5D-3L_GER_ and the depression scales (Table 2). To our knowledge, this is the first direct comparison of the convergent validity of the SF-6D and EQ-5D-3L in a population of patients with depressive symptoms. The picture that emerges in the literature from studies comparing either the SF-6D or the EQ-5D-3L with disease-specific instruments is rather heterogeneous. Brazier et al. summarized the existing evidence for mild to moderate depression and found values between |*r*_s_|= 0.35 and |*r*_s_|= 0.45 for the relationship between the EQ-5D-3L and HADS and values between |*r*_s_|= 0.56 and |*r*_s_|= 0.62 for the relationship between the SF-6D and the Clinical Outcomes in Routine Evaluation-Outcome Measure (CORE-OM) [17]. Peasgood et al. found that the EQ-5D-3L correlated well with measures of depression severity (|*r*_s_|= 0.54–0.77) [57]. In a recent study, the convergent validity of the SF-6D was evaluated using the Quality of Life Enjoyment and Satisfaction Questionnaire (Q-LES-Q) for depressive disorders. Good construct validity could be confirmed (|*r*_s_|= 0.74) [58].

Two possible explanations for the significantly better correlation of the SF-6D with disease-specific instruments compared to the correlation of the EQ-5D-3L with disease-specific instruments can be derived from the construction of the instruments. (1) The higher number of levels for the psychological dimension(s) of the SF-6D allows a more differentiated assessment of health states and particularly mild symptoms to be recorded more easily. However, since the low number of levels present in the EQ-5D-3L is a general problem that does not apply only to the psychological dimensions, the EuroQol group has since released an expanded version of the EQ-5D with five answer options (EQ-5D-5L). Abdin et al. investigated the convergent validity of the EQ-5D-5L using the Q-LES-Q for depressive disorders and determined good validity (|*r*_s_|= 0.67) [58]. Further research is needed to find out whether the validity of the EQ-5D-5L is really better than that of the EQ-5D-3L in depressed patients. (2) The EQ-5D focuses predominantly on the physical scope (four out of five dimensions), in contrast to the SF-6D, which is balanced between the physical and psychological scope with three dimensions each [19]. The weaker correlation of the EQ-5D-3L with the depression scales seems almost a logical consequence.

The predominant focus of the EQ-5D on the physical dimension of health might suggest that it is more suitable for use in somatic diseases than the SF-6D. Garcia-Gordillo and colleagues compared both instruments in a population of Parkinson’s patients and found almost identical, strong correlations with a disease-specific questionnaire [59]. A similar picture emerged for rheumatic diseases, with a slight advantage for the SF-6D (|*r*_s_|= 0.70 vs. |*r*_s_|= 0.80) [60]. In contrast, the EQ-5D-3L was shown to be more suitable for multiple sclerosis and non-specific back pain [61, 62]. The convergent validity of the EQ-5D-3L, therefore appears to be equally good or in some cases even better than that of SF-6D for somatic diseases.

### Internal responsiveness

The responsiveness of an instrument is of particular relevance in the context of health economic evaluations. If a HRQoL instrument is not responsive, a small but potentially clinically relevant change will not be reflected in the preference values and consequently not in the utility values (e.g. QALYs). Consequently, allocation decisions could be incorrectly influenced.

The SF-6D_UK_ is significantly more responsive in a population with depressive disorders than the EQ-5D-3L_UK_. The difference in responsiveness between the SF-6D_UK_ and EQ-5D-3L_UK_ is almost entirely due to the more than twice as high standard deviation of the EQ-5D-3L_UK._ A possible explanation from the different construction of the instruments has already been presented in the discussion on convergent validity. For both the EQ-5D-3L and the SF-6D, the responsiveness determined here was worse than that described in the literature for depressed or generally mentally ill people [63]. One possible reason for this could be the comorbid condition of the population, which influences the results of the generic index instruments differently than the sole presence of a mental illness.

For a population of mildly to moderately depressed patients, the literature describes effect sizes between − 0.68 and − 1.05 for the HADS, which are comparable to the present sample [17]. It is thus significantly more sensitive to change than the PHQ-9, for which only a small effect could be demonstrated. When comparing the disease-specific and generic instruments, the HADS was always the more responsive instrument. In contrast, the SF-6D_UK_ was significantly more responsive than the PHQ-9. The generally poorer responsiveness of the generic instruments, which has been repeatedly formulated but is also controversially discussed [8, 20, 21], could not be completely confirmed or refuted.

### External responsiveness

None of the AUCs generated from the ROC analysis reached the threshold of 0.70, which would be equivalent to a moderate ability to discriminate between patients with changed and unchanged depressive symptoms. In the context of mental disorders, only one other study was identified that found similarly poor AUCs for the EQ-5D-3L and the SF-6D in patients with schizophrenia [64]. However, the result of this study must be viewed critically, since only one ROC curve was generated for general change and not separately for improvement and deterioration as recommended in the literature [48, 49].

An important factor influencing the AUC is the choice of the external indicator criterion. In the present study, a change by the MCID of two points on the HADS was chosen for this purpose, as this was used as the primary outcome in the SPIRR-CAD study. In addition, the HADS is used in particular for the assessment of psychological stress in the context of somatic illnesses and thus seems suitable for the present population of patients with CAD and depressive symptoms. This choice might have been problematic for the determination of the external responsiveness of generic instruments. Generic instruments map not only the psychological dimension and its changes, but also those of the other components of quality of life that are likely to be influenced by CAD and other somatic comorbidities of the population under study. All dimensions influence the index value, which leads to an expected greater variance than on the HADS. However, in the absence of a gold standard for recording the HRQoL of mentally ill people, this is a general problem in quality of life research [17, 63].

### General aspects

Based on the results discussed so far, the SF-6D appears to be more valid and responsive than the EQ-5D-3L in patients with depressive symptoms. In addition to being more suitable for this specific population, the SF-6D as being derived from the SF-36 has another advantage. As a profile instrument, the SF-36 offers a detailed description of the individual dimensions of HRQoL and is therefore able to assess the consequences of an intervention in detail. The SF-36 is also widespread and often used in efficacy studies [8]. With its direct derivation from the SF-36 (or SF-12), the SF-6D offers the possibility to create a preference-based index value for cost–benefit analyses in the context of an effectiveness study without the need for an additional instrument.

### Limitations

The use of the SPIRR-CAD dataset for the methodological testing of the suitability of generic index instruments for capturing HRQoL of people with depressive symptoms is the key limitation. The population of patients studied had depressive symptoms as an inclusion criterion, but was simultaneously suffering from CAD, and many patients had additional comorbid illnesses. Thus, in contrast to the disease-specific instruments, the index scores of the generic instruments are not only influenced by the mental illness, but also by the limitations in the physical dimensions of the HRQoL caused by somatic comorbidity. The fact that the patients are also significantly impaired in the physical dimensions of quality of life is shown by the baseline value of 37.65 (compared to the mean value of a representative population sample standardised to 50) on the SF-36 physical health component score (PCS). This is lower than that of the SF-36 mental health component score (MCS) and also changes to a significantly lesser extent by the time of measurement after 18 months. The EQ-5D-3L may have been more influenced by the CAD and other somatic comorbidities than the SF-6D, as the former focuses predominantly on the physical dimensions, while the latter is balanced across the physical and mental dimensions.

Another limitation is associated with the value sets used. The choice of the value set for deriving the preference-based index has an impact on the result, as the preferences of the population of different countries may differ [65]. This is also very clear in this paper. The mean scores of the EQ-5D-3L_GER_ and EQ-5D-3L_UK_ differ greatly, while those of the EQ-5D-3L_UK_ and SF-6D_UK_ are relatively close. Based on these results, the decision to use the UK Value Set for hypothesis testing can be questioned. After all, German patients filled out the EQ-5D-3L and the SF-6D (via the questions of the SF-36) and the British index scores might misrepresent this self-assessment. Especially for a better comparability of the EQ-5D-3L and SF-6D (due to the fact that no German value set for the SF-6D exists) and because of the high correlation between the EQ-5D-3L_GER_ and EQ-5D-3L_UK_, this decision nevertheless appears to be justified.

## Conclusion

Both the convergent validity and the responsiveness of the SF-6D are better than those of the EQ-5D-3L in patients with depressive symptoms. Based on the evaluated data, the SF-6D therefore appears to be more recommendable than the EQ-5D-3L for use in studies to evaluate interventions for this population. With its consistently lower standard deviation and thus more accurate estimates, the SF-6D also appears to be a more suitable instrument for cost-effectiveness studies than the EQ-5D-3L. In this regard, it would be desirable for the German context to design and conduct a valuation study for the SF-6D.

### Supplementary Information

Below is the link to the electronic supplementary material.

Supplementary file1 (DOCX 16 KB)

## Data Availability

Not applicable.
