# Supplementary material for: Assessment of health-related quality of life in individuals with depressive symptoms: validity and responsiveness of the EQ-5D-3L and the SF-6D
Source: Eur J Health Econ. 2022 Nov 16;24(8):1297–307. doi: 10.1007/s10198-022-01543-w (PMC10533591; doi:10.1007/s10198-022-01543-w)
Supplement: Supplementary file 1 — Supplementary file1 (DOCX 16 KB) [file 10198_2022_1543_MOESM1_ESM.docx]

# Supplementary Information: Assessment of health-related quality of life in individuals with depressive symptoms: validity and responsiveness of the EQ-5D-3L and the SF-6D

**Table 1 Results of significance testing for hypothesis 1**

| **Comparison of correlation coefficients** | **Dunn and Clark’s z** | **P-value** |
| --- | --- | --- |
| Hypothesis 1: |  |  |
| rs (EQ-5D-3LUK – HADS) and **rs (SF-6DUK – HADS)** | -4.13 | <0.001* |
| rs (EQ-5D-3LUK – HADS) and **rs (SF-6DUK – PHQ-9)** | -4.87 | <0.001* |
| rs (EQ-5D-3LUK – PHQ-9) and **rs (SF-6DUK – HADS)** | -1.96 | 0.050 |
| rs (EQ-5D-3LUK – PHQ-9) and **rs (SF-6DUK – PHQ-9)** | -4.40 | <0.001* |
| r_s_: Spearman rank correlation coefficient  * significant for an α’ of 0.0125  The larger correlation coefficient is shown in **bold**. | | |

**Table 2 Results of the Modified Jacknife for hypotheses 2 and 3**

|  | **b_0_** | **99,375% CI** | | **SE** | **t** | **P-value** |
| --- | --- | --- | --- | --- | --- | --- |
|  |  | **Lower endpoint** | **Upper endpoint** |  |  |  |
| **SES** | | | | | | |
| EQ-5D-3L_UK_ und **HADS**^2^ | -0.859 | **-1.079** | **-0.723** | 0.054 | -16.787 | <0.001 |
| EQ-5D-3L_UK_ und **PHQ-9**^2^ | -0.308 | **-0.449** | **-0.145** | 0.052 | -5.860 | <0.001 |
| SF-6D_UK_ und **HADS**^2^ | -1.192 | **-1.380** | **-1.038** | 0.057 | -20.789 | <0.001 |
| **SF-6D_UK_** und PHQ-9^2^ | -0.582 | **-0.762** | **-0.413** | 0.057 | -10.248 | <0.001 |
| **SF-6D_UK_** und EQ-5D_UK_^3^ | 0.382 | **0.242*** | **0.512*** | 0.059 | 6.611 | <0.001 |
| **SRM** | | | | | | |
| EQ-5D-3L_UK_ und **HADS**^2^ | -0.424 | **-0.592** | **-0.271** | 0.055 | -8.016 | <0.001 |
| EQ-5D-3L_UK_ und **PHQ-9**^2^ | -0.376 | **-0.518** | **-0.221** | 0.051 | -7.309 | <0.001 |
| SF-6D_UK_ und **HADS**^2^ | -0.681 | **-0.839** | **-0.522** | 0.054 | -12.418 | <0.001 |
| **SF-6D_UK_** und PHQ-9^2^ | -0.635 | **-0.799** | **-0.492** | 0.054 | -11.898 | <0.001 |
| **SF-6D_UK_** und EQ-5D-3L_UK_^3^ | 0.361 | **0.224*** | **0.482*** | 0.053 | 6.646 | <0.001 |
| b_0_: intercept coefficient_;_ CI: confidence interval; SE: standard error; t: test statistics  *results for 97,5% confidence interval  ^2^ Comparisons for hypothesis 2  ^3^ Comparisons for hypothesis 3  Used „centered SES/SRM“ of second instrument respectively.  Confidence intervals and standard error determined per BCa-Bootstrapping with 1000 samples.  The more responsive instrument is shown in **bold**. | | | | | | |
